# Spider dung beetles: coordinated cooperative transport without a predefined destination

**DOI:** 10.1098/rspb.2023.2621

**Published:** 2024-01-17

**Authors:** Claudia Tocco, Marcus Byrne, Yakir Gagnon, Elin Dirlik, Marie Dacke

**Affiliations:** ^1^ Department of Biology, Lund Vision Group, Lund University, Lund, Sweden; ^2^ School of Animal, Plant and Environmental Sciences, University of the Witwatersrand, Johannesburg, South Africa

**Keywords:** insects climb in pairs, orientation precision of pairs, brood ball, mate choice, object transportation, *Sisyphus*

## Abstract

Cooperative transport allows for the transportation of items too large for the capacity of a single individual. Beyond humans, it is regularly employed by ants and social spiders where two or more individuals, with more or less coordinated movements, transport food to a known destination. In contrast to this, pairs of male and female dung beetles successfully transport brood balls to a location unknown to either party at the start of their common journey. We found that, when forced to overcome a series of obstacles in their path, transport efficiency of pairs of beetles was higher than of solo males. To climb tall obstacles with their common ball of dung, the female assisted the leading male in lifting the ball by steadying and pushing it upwards in a ‘headstand’ position during the climb initiation. Finally, we show that pairs were faster than single beetles in climbing obstacles of different heights. Our results suggest that pairs of *Sisyphus* beetles cooperate in the transportation of brood balls with coordinated movements, where the male steers and the female primarily assists in lifting the ball. Taken together, this is to our knowledge, the first quantitative study of cooperative food transport without a known goal to aim for.

## 1. Introduction

Intraspecific cooperative behaviour refers to collaborative actions by kin or unrelated individuals to assist others with the costs of the action, usually repaid by future fitness benefits [1]. These types of actions include babysitting activities of non-breeding mongoose [2], midwife assistance to fruit bats while giving birth [3], vigilance for predator detection in capuchin monkeys, Siberian jays and coral reef rabbitfish [4–6], as well as group hunting in ice killer whales [7], Harris' hawks [8] and wolves [9]. Energy saving strategies in fish schools, where individuals alternate between high cost and less costly positions, represent another type of intraspecific cooperation [10]. Here, we describe the collaborative transport behaviour of dung balls by pairs of small dung-beetle species.

Cooperative transport occurs when two or multiple individuals join forces to move an item between two sites and it often allows for the transportation of items well beyond the capacity of a single individual [11]. Social spiders, for example, move prey 700 times heavier than the dry weight of a single spider within their common web [12]. Cooperative transport is also regularly employed by ants, where cooperative movements of heavy food items can be either uncoordinated with individuals pulling in different directions, or coordinated by the ants encircling the food item, or by them all facing the same way [11,13]. It is important to note that each individual ant, or spider, involved in the cooperative transport of food strives towards the same final destination; either the nest, or a tightly spun shelter, in which to store the food. In this way, all the combined movements, coordinated or not, will gradually bring the food closer to this goal. In contrast to this, pairs of male and female dung beetles fluently collaborate to transport food to a location unknown to either party at the start of their common journey [14–17].

Ball-rolling dung beetles transport balls of dung and bury them in the soil, for feeding or breeding [16–18]. After carefully constructing a dung ball at the dropping site, these beetles immediately roll it away along a linear path to avoid intra- and interspecific competition for food and nesting sites [18–20]. This characteristic straight-line escape not only guarantees that the ball-rolling beetle will not inadvertently return to the competition at the dropping site, but also effectively maximizes the beetle's distance from it with every step taken [21]. The constant roll-bearing is maintained with the aid of the sun, or the moon, or the celestial polarization pattern or even the direction of the wind, depending on the activity period of the species and the habitat type [22–25]. It is important to note that a ball-rolling beetle does not set a goal destination at the beginning of its journey, but rather follows a set bearing until a suitable spot to bury its ball is encountered. Indeed, if the ball of a rolling beetle is stolen and the beetle is returned to the same dung pile again, the beetle makes a new ball and rolls it away along a different bearing [26]. When paired up for mating, the exact location at which the pair choose to stop and bury the brood ball is also selected on the go, again on the basis of the properties of the terrain being traversed [15–18].

Depending on the species, male and female beetles take part in the transportation of the brood ball with different degrees of involvement. In some of the large *Kheper* species*,* the female clings to the ball and lets herself be transported by the ball-rolling male [14,16]. Within the *Scarabaeus* species*,* we also find examples where the female simply walks behind the ball rolling male [14,16,17,27]. The male and the female will in this way reach a suitable nesting site together, but neither of these behaviours can be considered cooperative since the female performs no actions to assist the male in the ball-rolling process. However, females of Gymnopleurini and Sisyphini tribes show a higher degree of involvement, seemingly pushing or pulling the brood ball in collaboration with the male [14,15,17].

More than 100 years ago, Fabre [27] vividly described the transportation of a brood ball by a pair of *Sisyphus schaefferi* (Linnaeus). In their efforts to move the ball away from the dung pile, the pair of beetles repeatedly had to climb over obstacles so as to not deviate from their selected path. *Sisyphus* sp*.* are now known to use celestial cues to steer [28], but how this translates into cooperative transport, where both individuals ideally should direct their movement attempts in a common direction has never been addressed. Fabre [27] came to the conclusion that the female was the larger individual that held the ball with her front legs and pulled it while moving backwards. At the same time, Fabre thought the male pushed the ball in the same direction using his hind legs. More recent studies on this peculiar behaviour concluded that the pushing and pulling roles are sex-independent and that the pair even reverse their positions during the rolling process [15,17,29]. This raises further questions regarding how the transportation of the common brood ball is coordinated between the sexes.

Here, we studied the transport behaviour of brood balls by pairs of the Southern African *Sisyphus fasciculatus* Boheman and Palaearctic *S. schaefferi*. Both species are small in size and associated with woodland habitats [28,30,31], i.e. they commonly encounter obstacles on their rolling paths represented by plant material that typically accumulates on the terrain of their habitats. Cooperative transport of the brood ball could thus be beneficial for these small, closed habitat species. In this study we address the following questions: (i) can pairs of beetles cooperatively transport their common brood ball, i.e. is the transport efficiency greater when rolling in pairs rather than solo? if so, (ii) does working in pairs affect transport efficiency differently depending on the challenges encountered on the rolling paths (presence of obstacles and obstacles of different heights)? and finally, (iii) which transportation roles do male and female beetles take? We first assessed the effect of cooperative transport on speed and the tortuosity of rolled trajectories on flat terrain, and then when the beetles were forced to overcome obstacles in their path. We also quantified how solo beetles and pairs of beetles negotiated obstacles of different heights. We found that pairs of *Sisyphus* beetles do indeed cooperate in the transportation of brood balls, resulting in greater transport efficiency in the face of obstacles. Our results further suggest that this cooperation is driven by coordinated movements where the male steers while the female primarily assists in lifting the ball whenever obstacles need to be climbed. Taken together, this is to our knowledge, the first quantitative study of cooperative transport without a known goal to aim for.

## 2. Methods

### (a) Specimen collection and sex identification

Adult *S. fasciculatus* were collected in South Africa at Pullen Nature Reserve, Mpumalanga Province (25°34′01.6″ S, 31°10′42.5″ E) in November 2019 and January 2022. Adult *S. schaefferi* were collected near the Tempio di Antas–Sardus Pater, Province of Carbonia-Iglesias, Italy (39°23'25.6″ N, 8°29'15.8″ E) in June 2021 and May 2022. All beetles were collected using dung baited pitfall traps of the flat-bait trap type [32], each baited with 200 g of fresh cow dung.

Sexing of *S. fasciculatus* individuals used in the experiments was performed *in situ* using the presence (male) or absence (female) of a ‘comb’ of strong long setae on the ventral edge of the metatibia. The reliability of this method was confirmed by the extraction of the genitalia of 10 individuals used in the arena experiments. The absence of an obvious sexually dimorphic character in *S. schaefferi* was addressed by post hoc examination of the genitalia by dissection of all individuals tested.

### (b) Experimental set-up

Experiments were conducted outdoors under clear skies, at solar elevations between 20° and 60°; in Johannesburg, South Africa in December 2019 and in Vryburg, South Africa in February 2022 (January average temperature 19.7°C, humidity 68%; [33]) and in Gonnesa, Italy in July 2021 and May 2022 (June average temperature 23.1°C, humidity 53%; [33]), with *S. fasciculatus* and *S. schaefferi*, respectively. Each experimental session started with the transfer of approximately 50 beetles (average number of *Sisyphus* in a dung-trap collection [34]) into a plastic container (60 × 40 × 10 cm) with a 2 cm layer of sandy loam soil, and about 1 kg of fresh cow dung. Here, the beetles spontaneously formed pairs while constructing dung balls. A rolling pair consisted of a ‘dragger’ beetle and a ‘pusher’ beetle interacting with the same dung ball. Draggers were marked with a dot on their pronotum using a white marker (Tipp-Ex). Experiments involving ball rolling on flat terrain or climbing over obstacles were performed with one pair at a time, starting with the transfer of an actively rolling pair from the plastic container to the centre of a sand coated, circular, wooden, arena (30 cm radius) with or without obstacles set on its surface. Rolling paths were recorded using a Sony Handycam HDR-CX730E (fitted with a 0.42 × wide-angle lens) mounted from 1.5 m above. The same camera was used for recording the beetles' climbs.

#### (i) Transport behaviour of sisyphus on flat terrain

**Figure 1.**
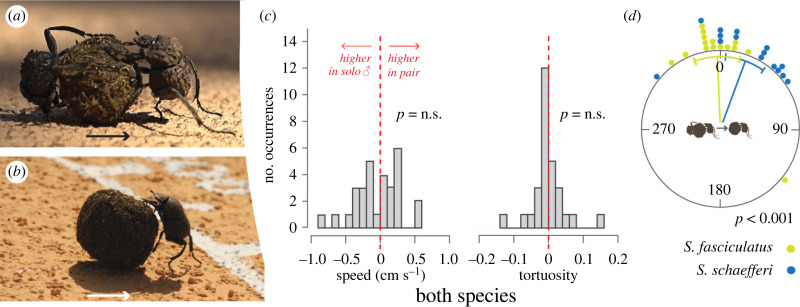
Transport efficiency of *Sisyphus fasciculatus* and *Sisyphus schaefferi* on a flat surface. For both species, 15 pairs and the 15 respective solo males were allowed to transport a dung ball from the centre to the perimeter of an arena. (*a*) Pair of *S. fasciculatus* and (*b*) male of *S. schaefferi* dragging a brood ball across the arena; arrows indicate the direction of travel. (*c*) Difference in speed and tortuosity (ratio between the length of the trajectory and the direct distance between its start and end point) between pairs and solo males, calculated as the difference in the mean of 10 rolls of the pair and the mean of 10 rolls of the respective male. For both species, generalized linear mixed models of speed and tortuosity showed no difference between pairs and males. (*d*) Change in bearing of pair and respective solo male. Green dot *S. fasciculatus*, blue dot *S. schaefferi*. For both species, V-test showed that the changes in bearing between the mean vector (µ) of the pairs and the males significantly cluster around 0°.

We first investigated if transport efficiency and bearing fidelity differed between pairs and solo individuals on even terrain. Fifteen pairs of each species were allowed to transport their shared ball from the centre to the perimeter of the flat arena (without obstacles) where the beetles’ exit bearing was recorded (figure 1*a*). The pair was carefully placed back at the centre of the arena and allowed to roll again for a total of 10 times. The ‘pusher’ was then separated from the ball and the solo ‘dragger’ returned with the ball to the centre of the arena where it was allowed to roll the ball to the perimeter an additional 10 times on its own (the pusher never rolled the ball further than 15 cm on its own).

#### (ii) Transport efficiency of pairs and solo individuals when negotiating obstacles

**Figure 2.**
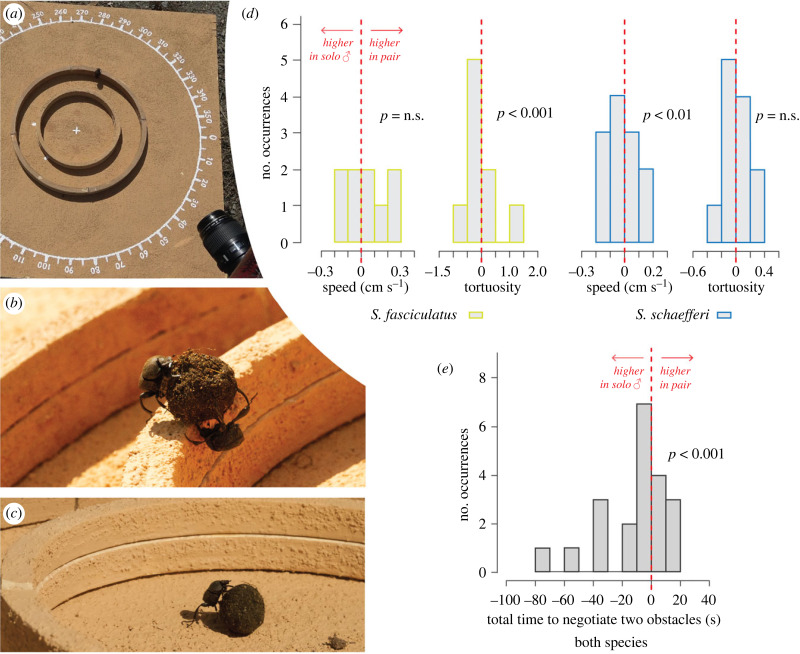
Transport efficiency of *Sisyphus fasciculatus* and *Sisyphus schaefferi* on the two-obstacles arena. For both species, 12 pairs and the 12 respective solo males were tested. (*a*) Arena with two obstacles: two rings (2.6 cm high) with inner radii of 8 cm and 16 cm. (*b*) Pair of *S. schaefferi* climbing the first obstacle; male in dragging position and female with its head and mesoleg on the obstacle. (*c*) Solo male dragging the pair brood ball and approaching the first obstacle. (*d*) Difference in speed and tortuosity between pairs and solo males for *S. fasciculatus* and *S. schaefferi*, and (*e*) difference in time taken from when beetles touched the first obstacle to when they fell down the far side of the second obstacle, calculated as the difference in the mean of the rolls of the pair and the mean of rolls of the solo male. Generalized linear mixed model (GLMM) with *post-hoc* analysis showed significant differences between pairs and solo males in tortuosity and speed in *S. fasciculatus* and *S. schaefferi*, respectively. For both species, GLMMs of total time to negotiate two obstacles showed differences between pairs and solo males.

In the next set of experiments, we evaluated the brood ball transport behaviour of *Sisyphus* pairs and solo individuals when obstacles were placed in their rolling path. Two concentric sand-coated plywood circular rings (height 2.6 cm, width 1 cm, inner radii of 8 cm and 16 cm) were added to the flat arena to form an obstacle course with two barriers for the beetles to climb sequentially (figure 2*a*). As for the flat arena trials, a rolling pair was randomly selected from the pairing box and allowed to roll their dung ball from the centre of the arena to its perimeter, where their exit bearing was recorded. This was repeated three times. Then, the ‘pusher’ was separated from the ball and the ‘dragger’ left to complete the same obstacle course solo, three more times. Twelve pairs were tested for both species.

#### (iii) How pairs and solo individuals climb obstacles of different heights

**Figure 3.**
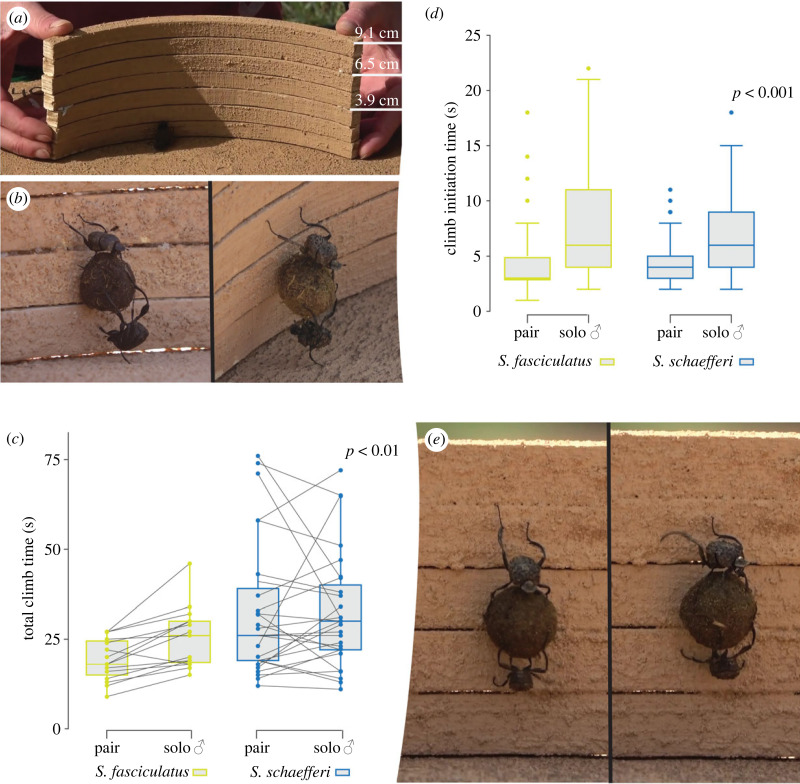
*Sisyphus fasciculatus* and *Sisyphus schaefferi* tested climbing sand-coated plywood obstacles of 3.9 cm, 6.5 cm or 9.1 cm height. (*a*) Plywood obstacles. (*b*) ‘Headstand’ of *S. schaefferi* and *S. fasciculatus* at the end of the climb initiation, i.e. from the moment when the beetle first made contact with the obstacle until the dung ball had been lifted from the ground and the leading male had reached a height of 2.6 cm high with the metatarsal claws of one leg. (*c*) Duration of the total climb for pairs and solo males of both species; grey lines link pair with its solo male. (*d*) Duration of the climb initiations ((see methods) of both successful and failed climbs) for pairs and solo males of both species. Generalized linear mixed models show significant differences between pairs and solo males in climb initiation time and entire climb time. Horizontal bars show the median and the boxes show the quantiles. (*e*) Pair of *S. fasciculatus* climbing a 6.5 cm obstacle; male in dragging position on the top and female in pushing position with the right mesoleg and head in contact with the obstacle.

To further evaluate the transportation abilities of beetle pairs versus solo males, for each species we tested 20 pairs and their respective solo males in a series of climbing experiments with increasing heights of obstacles. Again, an active pair was randomly selected and placed in the centre of the arena. The pair was allowed to roll for about 20 cm before a sand-coated plywood obstacle of 3.9 cm, 6.5 cm or 9.1 cm height (30 cm length, 1 cm wide) was placed perpendicular to the pair's rolling path (figure 3*a*). We allowed the beetles to initiate a climb over the obstacle up to three times for each obstacle height. Once all three obstacles had been presented to the pair, the ‘pusher’ was removed from the ball and the single ‘dragger’ was allowed to attempt to climb the same series of obstacles with the pair's ball but without the assistance of the ‘pusher’. For each obstacle height, we recorded the number of *climb initiations* and the number of *successful total climbs* as well as their duration. A *climb initiation* was defined as when a ‘dragger’ first made contact with the obstacle, then lifted the dung ball from the ground and reached 2.6 cm high with the metatarsal claws of one leg. According to this definition, the ‘pusher’, when present, was still in contact with the ground during the climb initiation. A *successful total climb* was defined as when the pair (or the solo ‘dragger’) reached the top of the obstacle and fell down the far side. We also calculated the ascending speed as the difference between the height of the climbing point (marked line on the obstacles at height of 3.9 cm, 5.2 cm 6.5 cm, 7.8 cm and 9.1 cm on the obstacles) reach by the dragger with the metatarsal claws of both legs and the height of 2.6 cm where the climbing initiation ended, divided by the elapsed time.

### (c) Data analysis

To evaluate if the dragger was the larger individual, as reported by Fabre [27], for each species, differences in body size (maximum pronotum width) between the male and the female in each pair were analysed for 15 pairs using a paired *t*-test. To study orientation precision of pairs and solo individuals, the mean vector and mean resultant vector length of 10 exit bearings was obtained using the package *circular,* v*.* 0.4–93 [35] and analysed using Prentice-Wilcoxon test (package *muStat,* v*.* 1.7.0; [36] and the V-test (package *CircStats,* v*.* 2–6; [37]). To calculate rolling speed (cm s^−1^) and tortuosity (the ratio between the length of the trajectory and the direct distance between its start and end point (dimensionless variable)), the trajectories of the beetles were automatically tracked using a custom-built auto-tracker (https://zenodo.org/records/10404735 in Julia language [38]).

Differences in dung-ball transport efficiency (speed and tortuosity of the roll trajectories, time to negotiate obstacles and ascending speed) between pairs and solo draggers were identified by fitting generalized linear mixed models (GLMMs) using the package *lme4* [39]. All fitted models accounted for fixed effects of condition (pair/solo dragger) and the pair's identifier was used as random effect to consider the two-block sampling design and handle the paired nature of the data. Each roll and climb event was defined as a sampling unit. A Shapiro-Wilk normality test was used to test for normality of dependent variables. Equidispersion of the count positive dependent variables was tested using the Overdispersion test (package *AER*; [40]). To confirm that skewness and kurtosis of all model residuals fell within the acceptable range (skewness between −1 and +1 and kurtosis between −3 and +3; [41] the package *phych,* v. 2.2.3 [42] was used.

#### (i) Transport efficiency models—full trajectories

As speed and tortuosity of the roll trajectories (dependent variables) were continuous, non-negative, and positive-skewed, a gamma family was specified in the models [43] and the heavily skewed tortuosity variables were also log-transformed. The Akaike information criterion, was used to identify the four models with the best fit to our data [44]. The best fit to speed and tortuosity on the flat arena was obtained without an interaction between condition (pair/solo dragger) and species (*S. fasciculatus*/*S. schaefferi*), while the best fit to speed and tortuosity on the two-obstacles arena was obtained with an interaction between condition and species (electronic supplementary material, table S1). *Post-hoc* Interaction analysis was used to calculate the pairwise differences between the condition and the species using the chi-square test (package *Phia* [45] and by correcting the *p*-values according to the Holm method.

#### (ii) Transport efficiency models—negotiation of obstacles

To identify possible differences in the time taken to negotiate the obstacles, we fitted GLMMs with the time (seconds) taken by the beetles to negotiate just the two obstacles and the space in between (‘ring travelling path’: inner radius 8 cm and outer radius 16 cm) as dependent variable. Normality and equidispersion of the dependent variable time were rejected and the negative binomial family was thus specified in the models [43]. To investigate if the ability of the single dragger to climb over the obstacles was affected by the number of rolling occasions we analysed the duration of the first, second and third occasion that each pair cleared the ring travelling path, as well as the duration of the first and the second occasion that a solo male cleared the same two-obstacle section on his own. This was done by fitting models with duration to negotiate the two-obstacle section as dependent variable and roll occasion as fixed effect.

#### (iii) Transport efficiency models—the effect of obstacle height

Possible differences between pairs and draggers in dung-ball transport efficiency as a consequence of the height of the obstacle cleared were defined by fitting GLMMs with the time taken to perform the climb initiation, duration for the total climb, and the ascending speed as dependent variables. In the model of total climb time, the interaction between the fixed effect and the obstacle height was also specified. Normality of all dependent variables were rejected, and equidispersion was assessed only for the duration of climb initiation. In this way, the Poisson family was specified in the climb initiation time model (count, positive, equidispersed variable), a gamma family in the ascending speed model (positive continuous variable), and a negative binomial family in the total climb model (count, positive, overdispersed variable) [43].

All models were analysed and developed in R 4.1.2 [46].

## 3. Results

After having constructed a small ball of dung (table 1), pairs of *S. fasciculatus* started to move their shared dung ball away for burial and presumably egg laying. The transportation strategy of the dung ball was identical for the two species: one beetle in each pair, the ‘dragger’, pulled the ball with its front legs while facing the ball and moving backwards, while the second beetle, the ‘pusher’, simultaneously appeared to push the ball in the same direction by the use of its back legs (figure 1*a*). Close examination of all pairs used in this study (47 *S. fasciculatus,* 47 *S. schaefferi*), revealed all draggers to be male and all pushers to be female. Males of both species were significantly larger than their companion females ((table 1), paired *t*-test_pronotum width_: *S. fasciculatus*: *t*_14_ = 2.47, *p* < 0.05, *S. schaefferi*: *t*_14_= 3.92, *p* < 0.01). It should be noted that while the male draggers could be encouraged to continue to roll the ball after the removal of the female (figure 1*b*), solo female pushers would at best roll the ball for 15 cm after removal of their partner, after which she would abandon the ball or sit motionless on top of it. Some of these motionless females were ‘reactivated’ by making the ball vibrate and only kept moving if the ball continued to vibrate and move. As a consequence, individual performance could only be measured for the male draggers.

**Table 1.** Mean and standard deviation of parameters and variables of *Sisyphus fasciculatus* and *Sisyphus schaefferi* considered in the study.

		*Sisyphus fasciculatus*		*Sisyphus schaefferi*	
ball measurements	diameter (mm), *n*= 15	13.5 ± 0.2		14.1 ± 0.9	
	weight (g), *n* = 15	1.0 ± 0.1		1.7 ± 0.3	
		male	female	male	female
body measurements	pronotum width (mm), *n* = 15	5.3 ± 0.3	5.1 ± 0.3	6.2 ± 0.39	5.7 ± 0.5
	weight (g), *n* = 15	0.13 ± < 0.1	0.12 ± < 0.1	0.15 ± < 0.1	0.13 ± < 0.1
		pair	male	pair	male
flat arena	speed (cm s^−1^), *n* = 15	1.3 ± 0.3	1.3 ± 0.5	1.5 ± 0.4	1.5 ± 0.3
	tortuosity, *n* = 15	1.0 ± 0.1	1.0 ± 0.1	1.03 ± 0.09	1.13 ± 1.11
	mean vector length (*R*), *n* = 15	0.94 ± 0.06	0.95 ± 0.06	0.93 ± 0.14	0.92 ± 0.06
two-obstacles arena	tortuosity, *n* = 12^a^	1.27 ± 0.59	1.27 ± 0.58	1.09 ± 0.14	1.09 ± 0.24
	speed (cm s^−1^), *n* = 12^a^	0.76 ± 0.29	0.78 ± 0.22	0.53 ± 0.16	0.53 ± 0.20
	ring travelling path (s), *n* = 12^a^	34.58 ± 28.05	42.05 ± 34.30	34.39 ± 16.57	46.74 ± 44.80
climbing experiment	ascending speed (cm s^−1^), *n* = 20	0.20 ± 0.19	0.15 ± 0.09	0.17 ± 0.13	0.18 ± 0.12
	climb initiations (s), *n* = 20	4.37 ± 2.85	7.75 ± 5.42	4.46 ± 2.20	6.91 ± 3.70

^a^Nine solo males of *S. fasciculatus*. Ring travelling path: two obstacles and the space in between. See methods section for details.

### (a) Cooperative transport behaviour on flat surfaces: actively steering male and well-coordinated female

We did not observe any significant differences in rolling speed between the species or between pairs and solo males (figure 1*c*), when rolling their dung balls on the flat arena (speed (table 1), GLMM, pair-male: est. = 0.02, *z*-value = 0.98, *p* = 0.33, *n* = 577). This held true also for the tortuosity of rolling paths, travelled across the flat arena (tortuosity (table 1), GLMM, pair-male: est. = −0.05, *z*-value = −0.35, *p* = 0.73, *n* = 577; figure 1*c*). To further evaluate the beetles' ability to adhere to a single bearing, we examined the orientation precision for pairs and solo males of both species, calculated as the mean vector length (R) for 10 exit bearings at the arena perimeter. Again, we did not find any significant differences in performance between pairs and solo males (*R* (table 1), Prentice-Wilcoxon test: *S. fasciculatus*: *Χ*_1_^2^ = 1.67, *p* = 0.20, *n* = 15; *S. schaefferi*: *Χ*_1_^2^ = 3.27, *p* = 0.07, *n* = 15). In summary, this means that neither transport efficiency nor orientation precision were positively—or negatively—affected by constant contact with the female when moving the common dung ball over the flat surface. This suggests well-coordinated behaviour by the female.

As indicated by a significant clustering around a 0° change in bearing between the mean vector (µ) of the pairs and the males (V-test: *S. fasciculatus*: µ = 4.71, *p* = 1.51^−7^, *n* = 15, *S. schaefferi*: µ = 4.59, *p* = 2.00^−7^, *n* = 15), the single male continued to roll along the same bearing even if the female had been removed from the ball (figure 1*d*). This clearly demonstrates the male had registered the bearing travelled by the pair. Whether this held true also for the female could not be tested as she refused to roll once the male had been removed.

### (b) Cooperative transport behaviour in response to obstacles increases transport efficiency

Next, to evaluate the effect of collaborative transport in a slightly more demanding orientation task, two obstacles (two rings (2.6 cm high) with inner radii of 8 cm and 16 cm) were added to the flat arena (figure 2*a*–*c*). We aimed to record a full series of three exits by 12 pairs and the respective solo male of both species of beetles. However, only five and seven of the males of the *S. fasciculatus* and *S. schaefferi* pairs (figure 2*b*) respectively completed a full series of three exits when challenged to transport the ball solo over both obstacles. Another three, and one solo male(s) of *S. fasciculatus* and *S. schaefferi* (figure 2*c*) respectively, exited the arena twice, while one, and four male(s) of *S. fasciculatus* and *S. schaefferi* respectively, exited the arena only once. This indicates that the dragging males were more reluctant and/or less capable to climb obstacles on their own, as compared to when the females pushed, or at least followed the ball with their hind legs.

When forced to negotiate the two obstacles on their paths from the centre to the perimeter of the arena (figure 2*a*–*c*), differences in transport efficiency (speed and tortuosity) between pairs and solo males, and between species (significant interaction between the fixed effect of condition (pairs/male) and the species) were revealed. The tortuosity of the paths travelled by pairs of *S. fasciculatus* was lower than for solo males, while there was no significant difference in speed between pairs and solo males’ (tortuosity (table 1), post-hoc analysis, pair-male: *Χ*_1_^2^ = 24.89, *p* < 0.001, *n* = 115; speed (table 1), post-hoc analysis, pair-male: *Χ*_1_^2^ = 0.03, *p* = 0.37, *n* = 115; figure 2*d*). The opposite holds true for *S. schaefferi,* where pairs were significantly faster than solo males in negotiating the two-obstacles' course, with no significant difference in tortuosity (tortuosity (table 1), post-hoc analysis, pair-male: *Χ*^2^_1_ = −0.44^−2^, *p* < 0.01, *n* = 115; speed (table 1), post-hoc analysis, pair-male: *Χ*^2^_1_ = −0.70, *p* = 0.06, *n* = 115; figure 2*d*). It is important to note that while the mean speeds are remarkably similar between the pairs and solo male beetles, the pairwise comparison clearly revealed that the tortuosity of the paired rolls of *S. fasciculatus* was significantly lower, and the speed significantly greater than when the male was left to negotiate the obstacle-course on his own (figure 2*d*).

To better understand the effect of obstacles on transport efficiency, we next focused our analysis on the time taken from when beetles touched the first obstacle to when they fell down the far side of the second obstacle. We now found that pairs of both species were significantly faster in clearing the two obstacles than their respective males rolling alone ((table 1), GLMM, pair-male: est. = −0.33, *z*-value = −3.41, *p* < 0.001; *n* = 112; figure 2*e*).

To further evaluate how the number of rolling occasions affected the ability of the beetles to climb over obstacles, we also analysed the time taken to clear the two-obstacle section of the arena, the first, second and third time within a pair, as well as the duration of the first and second climb of the solo males. The resulting consistency in time taken to clear the two-obstacle section indicates the absence of a learning or fatigue effect when repeatedly clearing these obstacles, (GLMM, roll1-roll2_pair_: est. = 0.05, *z*-value = 0.46, *p* = 0.64; roll1-roll3_pair_: est. = 0.01, *z*-value = 0.09, *p* = 0.93; *n* = 63; roll1-roll2_solo male_: est. = −0.02, *z*-value = −0.10, *p* = 0.92; *n* = 32).

### (c) Climbing strategy

Pairs of both species seemed to use the same climbing strategy to overcome tall obstacles. As soon as the dragger (moving backward) came into contact with an obstacle, he touched it repeatedly, searching for a grip with the claws on his metalegs. When a secure grip was found, the dragger pulled himself up from the arena floor with the metalegs and lifted the ball clear of the floor. According to our definition, when the dragger reached 2.6 cm high with one of the metalegs the climb initiation was completed (figure 3*b*). When present, the female assisted in lifting the ball by pushing it against the wall and upwards. She then positioned herself under the ball, with the dorsal part of her head and prothorax in contact with the floor, pushing and steadying the ball. From this position, she usually appeared to push the ball upwards and would finally perform a ‘headstand’ with only one leg on the floor (figure 3*b*). Alternating the grip between his left and right metalegs, the male then climbed up the obstacle while dragging the ball, and eventually also the female, with him. The female seems to simply follow this climbing process, occasionally touching the obstacle with a leg or her head. When at the top of the obstacle, the female became more active again, pushing against the obstacle with her head, assisting the final drop, down the far side of the obstacle.

It should be noted that when clearing tall obstacles, climbing head down with his metalegs leading on the chosen path, but only attached to the substrate with his metatarsal claws, the male ‘dragging’ beetle is transporting a ball that weighs 7 (*S. fasciculatus*) or 11 times (*S. schaefferi*) his body weight, in the vertical plane. When a female is also attached to the ball an additional 90% of the male's body weight is added to that load (table 1).

### (d) Pairs are more inclined and faster in negotiating higher obstacles than solo males

To further zoom in on the nature of the cooperative behaviour between the male and the female beetles, we gradually increased the difficulty of their straight-line ball transport by placing obstacles of three different heights in their way. When presented with a 3.9 cm high obstacle on three consecutive occasions, the 40 pairs of beetles (20 *S. fasciculatus* and 20 *S. schaefferi*) performed 47 climb initiations (when the metatarsal claws of at least one leg of the dragger reached a height of 2.6 cm) and succeeded in clearing the obstacle 37 times, i.e. with a success rate of 79%. When the females were removed, the climb initiations shown by the solo males decreased to 40 with a similar success rate of 73% (29 successful climbs). Increasing the height of the obstacles to 6.5 cm, the pairs initiated 49 climbs with a decreased success of 59% (29 successful climbs). The number of climb initiations by solo males for the same object dropped to 23, with a similarly decreased success of 61% (14 successful climbs). A further increase of the height of the obstacle to 9.1 cm, revealed a greater difference in how inclined the pairs were to initiate a climb (21 climb initiations with 48% success) compared to solo males (eight climb initiations with 63% success). It is interesting to note that while a solo male was less likely to attempt to climb a higher obstacle, his success was the same as for the second highest obstacle once the climb was initiated.

A closer analysis of the time taken for an entire climb—from when a beetle first made contact with the barrier, to when the beetle or beetles fell down the far side of the obstacle—revealed that males were significantly slower on their own (GLMM, total climb time, pair-male: est. = −0.18, *z*-value = −2.84, *p* < 0.01; *n* climbs = 84; figure 3*c*). To our surprise, we did not find any difference in ascending speed (of both successful and failed climbs) between pairs and solo males (ascending speed (table 1), GLMM, pair-male: est. = −0.02, *t*-value = −0.09, *p* = 0.93; *n* = 231). This meant that any differences observed in the duration of the climb, must be owing to differences at the very start of the climb. The duration of the climb initiations (of both successful and failed climbs), i.e the time from the moment when the beetle first made contact with the obstacle until the dung ball had been lifted from the ground and the leading male had reached a height of 2.6 cm high with the metatarsal claws of one leg, was significantly shorter when executed by a pair rather than by a solo male (climb initiation time (table 1), GLMM, pair-male: est. = −0.47, *z*-value = −9.82, *p* < 0.001; *n* = 302; figure 3*d*).

## 4. Discussion

In this study, we found that pairs of *Sisyphus* cooperate in the transport of their brood balls and do so in a coordinated way with defined roles and changes in behaviour based on the difficulty of the path travelled. However, unlike several ant species that cooperate in the transportation of food to a common nest [11,47], the *Sisyphus* pair rather roll their ball towards an unknown destination until they encounter suitable terrain in which to bury it [15]. To succeed in this, the male and female beetle have to closely coordinate the direction in which to push their brood ball, as pushing and pulling in different directions would negate straight-line efficient escape from the busy dung pat.

### (a) On flat terrain, the male steers and the female follows

When quantifying the paths travelled by pairs and solo males on a flat surface free of obstacles, we found no differences in transport efficiency (speed, tortuosity or orientation precision; figure 1*c*). That is, the male beetles pulled the ball and steered it along a chosen bearing with the same efficiency as when accompanied by the female. In addition, when the female was removed from the rolling pair, the solo male continued to move the brood ball along the same bearing. Because all solo females stopped rolling when the male was removed, the same analysis could not be performed for her performance, but her marked reaction to his sudden absence nevertheless suggests that she closely coordinates her movements with his; when he stopped moving the ball, she did the same. It is further interesting to note that while rolling as a pair, the presence of the female neither improved nor decreased the transport efficiency compared to that of the solo male. The female did not appear to steer the dung ball in a conflicting direction nor contribute to its transport with added force in the common direction. Consequently, on flat terrain, we interpret the female's contribution to the transportation of the brood ball as passive cooperation.

### (b) When negotiating obstacles, the female and the male coordinate the vertical transport of the brood ball

When two obstacles of 2.6 cm height were added to the arena in such a way that beetles were forced to climb them to transport their ball from the centre to the edge of the arena, pairs of beetles now performed better than solo males. The trajectories of *S. fasciculatus* pairs were straighter and the *S. schaefferi* pairs were faster than their respective lone males (figure 2*d*). This indicates that the female switched from a passive follower to an active helper to negotiate the two-obstacles' course presented to them. In addition, every pair of both species completed a full series of three exits, while many solo males only cleared the obstacles once or twice before abandoning the ball and the experiment. Both species, *S. fasciculatus* and *S. schaefferi* share a preference for woodland and forest habits [28,30,31], frequently having to roll their dung balls over uneven terrain covered with plant litter. Cooperative transport may very well be the solution adopted by *Sisyphus* species to transport their brood balls over such difficult ground. It could also help in assessing mate quality [48].

At first sight, it may appear easier if the female simply walked behind the ball—as observed in some *Scarabaeus* spp*.* [13–17] and *Circellium bacchus* [49]—instead of following it backwards, head down and with her back legs moving over the tumbling brood ball. Like ants relying on points of contact with the object to gain information about the forces applied by other individuals [47], the tactile interaction with the ball might very well help the female to coordinate her movements with the male and to quickly assist him when needed. Maintaining contact with the ball could also potentially be a form of mate-guarding, but as female mate-guarding has been proved only among polygynous birds [50,51], and we have not seen any other behaviour to support this suggestion, we consider this unlikely.

### (c) The female assists in the initiation of a climb

To more closely define the nature of the cooperation between the male and the female beetles, we gradually increased the difficulty of their straight-line ball transport by placing obstacles of three different heights (3.9 cm, 6.5 cm or 9.1 cm) in their path. We found that the higher the obstacle, the fewer attempts were made by the pairs and solo males to climb it. The number of attempts made, however, decreased more rapidly for solo males compared to pairs. It is interesting to note that the success rate for the solo males that did initiate a climb was similar to that of the pairs. This indicates that a male can clear even the highest obstacle with the dung ball on his own, but that this is probably less rewarding. Because the female actively grips the barrier as she ascends with the male and the ball, she provides an extra anchorage point. Indeed, the male, holding the ball with four legs and climbing with his two hind legs, frequently gripped the wall with only one metatarsal claw. Like two climbers belaying each other on a wall, we repeatedly observed females preventing a fall when the male temporarily lost his grip on the wall. Had he been alone, the male would have had to start again. To our surprise, the ascending speed of the pair was not greater in the presence of a female, who thus seemed to provide assistance only when critically needed.

Nevertheless, we also found that the total time taken to climb the obstacles was significantly shorter for the pair than for the solo male. While this at first seemed contradictory to the similar ascending speeds identified above, the answer can be found in the significantly shorter duration of the initiation of the climb by a pair (*S. fasciculatus*: 4.4 s versus 7.8 s; *S schaefferi*: 4.5 s versus 6.91 s; figure 3*c*). The start of the climb was defined from the moment a ‘dragger’ collided with the obstacle until the dung ball was lifted from the arena *and* the leading beetle reached a height of 2.6 cm with the metatarsal claws of one leg. These two events often coincide in time. A close inspection of the actions of the female when initiating a climb over the vertical objects together with the male, revealed that the female positioned herself under the ball, stabilizing it with her legs while pushing it up in unison with the climbing male who was already vertically placed on the wall. The faster climb initiation, and thus swifter clearing of the obstacle (figure 3*d*) can probably be attributed to this characteristic female ‘headstand’. Again, for this action to be beneficial, the female needs to coordinate her movements of the ball with those of the male (figure 3*e*). This collaborative behaviour for overcoming obstacles is extremely beneficial if we consider that both *Sisyphus* species roll over litter-strewn terrain, typical in the closed habitats where they occur [15,30,31]. *Sisyphus* are also relatively small dung beetles, taking short steps and therefore prone to travelling along more tortuous paths [21], consequently encountering more obstacles. Indeed, it has been reported that beetles with a similar step length to *S. fasciculatus* bury their dung ball at slightly closer distance to the dung pat compared to individuals of a larger species (*Scarabaeus ambiguus* 7 m; *Kheper lamarcki* 12 m), however, the total distance rolled by the smaller species was greater owing to the greater tortuosity of its path [21].

### (d) Why does the male lead ball transport while the female follows?

In this study, we identified all ball dragging beetles as male and all pushing beetles as female. This sex dependent positioning for ball transport stands in contrast to earlier studies of this behaviour. Fabre [27] attributed the ‘mother role’ to the slightly larger individual of the pair that was in the ‘place of honour’ in front. In this study, we found the males of both species to be bigger than the females and to be leading. Consequently, according to our identification, the larger male drags from the front of the balls' path. Paschalidis [15] and Rizzotto *et al*. [29] report that the male usually pushes and the female drags (i.e opposite to what we found), but in those studies the sex was determined by external morphological characters, which can be tricky in these small beetles. Based on our strict sex identification criteria (extraction of genitalia), we are confident in our claim that it is the female, that in other genera conserves her energy by clinging to the brood ball or walking close to it [14,16,17,27], that takes the more passive role and only actively assists the male in the transportation of the brood ball when assistance is needed.

This is, to our knowledge, the first quantitative study in non-human animals of a cooperative coordinated transport without a known final destination. To ensure smooth and effective transport, efficient communication has to take place between male and female of the beetle pair. However, the mechanism that allows male and female of the beetle pair to communicate and coordinate their joint actions is currently not known. The fact that, when the male is removed from the brood ball, the female becomes inactive, but she can be ‘reactivated’ by making the ball vibrate suggests that mechanoreceptor rather than vision or olfaction are involved in the communication process. This topic remains open to further investigation.

## Data Availability

Data supporting this article are available as the electronic supplementary material [52]. Custom-built auto-tracker available at https://zenodo.org/records/10404735 [53].
